# Sex‐Specific Long‐Term Effects of Perinatal *Limosilactobacillus reuteri* on Social Cognition, Gene Expression, and Gut Microbiota

**DOI:** 10.1111/jnc.70199

**Published:** 2025-08-19

**Authors:** Tatiana Siegler Lathrop, Inés Martínez Sanchez, Ioannis S. Chronakis, Rochellys Diaz Heijtz

**Affiliations:** ^1^ Laboratory of Nano‐BioScience, DTU‐Food, Research Group for Food Production Engineering Technical University of Denmark Lyngby Denmark; ^2^ Department of Neuroscience Karolinska Institutet Stockholm Sweden

**Keywords:** early‐life, interleukin‐10, maternal probiotics, prefrontal cortex, social behavior, striatum

## Abstract

Recent research highlights the potential of early‐life probiotic interventions to promote brain health later in life. In this study, we investigated the long‐term effects of *Limosilactobacillus reuteri* ( *L. reuteri* ) supplementation during a critical perinatal window (gestational Day 6 to postnatal Day 7) on behavioral, molecular, and gut microbiota outcomes in adult male and female BALB/c mice. Perinatal *L. reuteri* supplementation led to significant and lasting improvements in sociability, social recognition, and gut microbiota composition in male offspring. These changes were accompanied by increased gene expression of the anti‐inflammatory cytokine *Il10* in both the striatum and colon of male offspring. Notably, expression of the oxytocin receptor (*Oxtr*), a key regulator of social and anxiety‐like behaviors, was significantly increased in both the prefrontal cortex and striatum in males and females. However, probiotic‐exposed females exhibited a distinct behavioral profile, showing a trend toward reduced anxiety‐like behavior but impaired social recognition. They also displayed increased gene expression of the peptidoglycan transporters *Slc46a2* and *Slc46a3* in the striatum, whereas only *Slc46a2* was elevated in males, suggesting a potential mechanistic pathway underlying the observed sex‐dependent effects. These findings indicate that perinatal *L. reuteri* supplementation modulates the microbiota–gut–brain axis in a sex‐specific manner, influencing behavior, neuroimmune signaling, and gut microbiota composition. Our results underscore the importance of accounting for sex differences when developing early‐life microbiota‐based interventions for neurodevelopmental disorders and long‐term brain health.

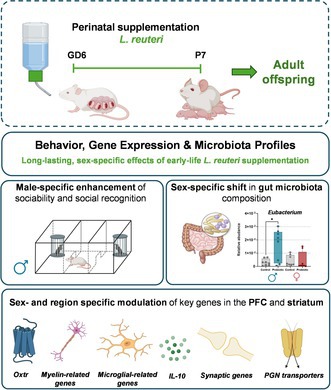

## Graphical abstract

Perinatal supplementation with *Limosilactobacillus reuteri* ( *L. reuteri* ) during gestation and early postnatal life induces long‐lasting, sex‐specific effects on the microbiota–gut–brain axis. Treated male offspring showed enhanced sociability, social recognition, microbial shifts, and coordinated transcriptional changes across the brain and gut. Female offspring did not exhibit similar behavioral benefits but showed selective gene expression changes. These findings underscore the potential of early‐life microbial interventions to support social functioning and highlight sex differences relevant to future strategies for neurodevelopmental disorders such as autism spectrum disorder.

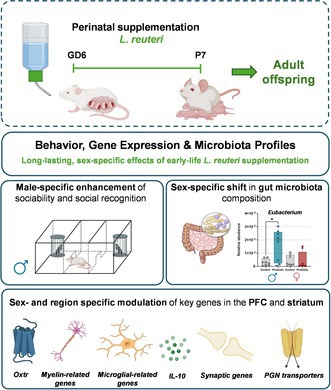

**Abbreviations**
ASD: autism spectrum disorder
Avpr1a: arginine vasopressin receptor 1a
Avpr1b: arginine vasopressin receptor 1b
Bdnf: brain‐derived neurotrophic factor
Cd11b (Itgam): cluster of differentiation 11b
Cldn3: claudin‐3
Cx3cr1: CX3C motif chemokine receptor 1
3‐CST: three‐chamber social approach task
Darpp32 (Ppp1r1b): dopamine‐ and cAMP‐regulated phosphoprotein 32
EPM: elevated plus maze
F11r: junctional adhesion molecule A (JAM‐A)
GD: gestational day
Il10: interleukin‐10
Il6: interleukin‐6
Iba1 (Aif1): ionized calcium‐binding adaptor molecule 1
LDB: light–dark box

*L. reuteri*

: *Limosilactobacillus reuteri*
Mag: myelin‐associated glycoprotein
Mbp: myelin basic protein
Mog: myelin oligodendrocyte glycoprotein
Muc3: mucin 3
Ocln: occludin
Oxtr: oxytocin receptor
P: postnatal day
PFC: prefrontal cortex
SCFA: short‐chain fatty acids
STR: striatum
Syp: synaptophysin
Tjp1: tight junction protein 1 (ZO‐1)
Trem2: triggering receptor expressed on myeloid cells 2

## 1 Introduction

Over the past few decades, the microbiota–gut–brain axis—the bidirectional interaction between gut microbes and the brain—has been identified as a key regulator of brain development, function, and behavior. Foundational studies using germ‐free (GF) mice and antibiotic‐treated animal models have demonstrated that the gut microbiota profoundly influences early‐life brain programming, affecting key brain functions such as cognition, emotional regulation, and motor control (Cryan et al. 2019). It is well established that the maternal environment during pregnancy is critically important for offspring brain development, shaping long‐term neurodevelopmental trajectories and susceptibility to mental health disorders across the lifespan (Al‐Haddad et al. 2019; Codagnone et al. 2019). Extensive research has revealed that common environmental risk factors—such as stress, inflammation, diet, and antibiotic use—affect the maternal gut microbiota, indicating that the microbiota serves as a key shared pathway through which these factors influence neurodevelopment (Bolte et al. 2022; Catassi et al. 2024). Given this growing recognition, maternal microbiota‐targeted interventions—such as probiotic supplementation—are gaining traction as novel strategies to promote healthy neurodevelopment and behavior, while potentially reducing the risk of neurodevelopmental conditions (Siegler Lathrop et al. 2024; Cuinat et al. 2022).

Among single‐strain probiotics, *Limosilactobacillus reuteri* ( *L. reuteri* ) has shown great promise in modulating brain social networks (Weber et al. 2024). This Gram‐positive bacterium, naturally present in both human and rodent gut microbiomes, supports gut health by producing antimicrobial compounds such as reuterin, promoting anti‐inflammatory cytokine expression at both the mRNA and protein levels in experimental models, enhancing mucosal barrier integrity, and stimulating IgA production to help regulate the intestinal microbial community (Abuqwider et al. 2022; Mu et al. 2018; Peng et al. 2023). Studies have demonstrated that postnatal *L. reuteri* supplementation rescues social deficits in multiple autism spectrum disorder (ASD) mouse models—including genetic (Shank3B deficient), environmental (valproic acid, GF, maternal high‐fat diet), and idiopathic (BTBR T+ Itpr3tf/J) models (Buffington et al. 2016; Sgritta et al. 2019). These effects occur, at least in part, via mechanisms involving the vagus nerve and the oxytocin–dopamine reward system (Sgritta et al. 2019). Additionally, maternal *L. reuteri* administration during lactation normalizes blood–brain barrier function and improves cognitive outcomes in offspring exposed to prenatal lipopolysaccharide (Lu et al. 2023). Beyond neurodevelopment, *L. reuteri* exhibits anti‐inflammatory properties in autoimmune models such as experimental autoimmune encephalomyelitis, where it alters gut microbiota composition and reduces pro‐inflammatory responses (He et al. 2019). In pregnant Sprague–Dawley rats, maternal *L. reuteri* supplementation during pregnancy ameliorates high‐fat diet‐induced programmed hepatic steatosis in the offspring, as well as improves placental remodeling and reduces oxidative injury (Yu et al. 2022). A key mechanism underlying these effects is the ability of the probiotic to enhance oxytocin signaling, as *L. reuteri* stimulates oxytocin secretion from intestinal enterocytes via secretin signaling (Danhof et al. 2023). Given that oxytocin is essential for neurodevelopment, particularly in the formation of social behaviors and cognitive functions (Glasper and Neigh 2020; Varian et al. 2023), these findings suggest that perinatal *L. reuteri* supplementation may have long‐lasting neurobehavioral consequences on offspring.

Clinical trials have also begun to explore the therapeutic potential of *L. reuteri* for neurodevelopmental and metabolic conditions. In children with ASD, a product containing a combination of the *L. reuteri* strains ATCC‐PTA‐6475 and DSM‐17938 significantly improved social functioning, but not autism severity (Mazzone et al. 2024). Similarly, treatment with SB‐121 (a combination of *L. reuteri* , dextran microparticles, and maltose) was associated with improvements in adaptive behavior and social preference in adolescents and adults with ASD, as well as increased oxytocin levels (Schmitt et al. 2023). Additionally, supplementation with *L. reuteri* LR‐99 in individuals with Prader‐Willi syndrome enhanced social behaviors, fine motor skills, and reduced BMI, suggesting broader relevance beyond ASD (Kong et al. 2021). Extending to other health domains, *L. reuteri* has also been investigated in prenatal clinical trials for allergy prevention. Maternal *L. reuteri* supplementation, followed by lactational exposure in offspring, led to epigenetic modifications in CD4+ T cells that may influence immune maturation and allergy susceptibility (Forsberg et al. 2020). However, a major limitation of clinical studies related to ASD is that research outcomes have primarily focused on males, leaving open the question of whether both sexes derive equal benefits from early‐life *L. reuteri* interventions.

Here, we investigated the long‐term effects of perinatal *L. reuteri* supplementation—administered during the critical developmental window from gestational day (GD) 6 to postnatal day (P) 7—on social cognition, anxiety‐like behavior, gene expression, and gut microbiota composition in both male and female offspring. This time window is particularly relevant as it overlaps with the development and maturation of the oxytocin system (Grinevich et al. 2014), a critical system responsive to *L. reuteri* supplementation. Importantly, we used BALB/c mice, a strain that exhibits atypical social behaviors, including reduced sociability (Brodkin 2007), making them well suited for testing pro‐social interventions. By leveraging this model, we aimed to determine whether perinatal exposure to *L. reuteri* could modulate sex‐specific neurodevelopmental and microbial outcomes, providing novel insights into precise microbiota‐based strategies for early‐life intervention and potential prevention.

## 2 Material and Methods

### 2.1 Animals

Time‐mated pregnant BALB/cJRj mice were obtained from Janvier Labs and housed individually in Makrolon Type III polycarbonate cages with standard bedding and nesting material. Mice were maintained under controlled environmental conditions (temperature: 22°C ± 1°C; humidity: 55% ± 10%) on a 12:12‐h light–dark cycle. The BALB/cJRj strain was selected for its characteristically low baseline sociability and heightened stress sensitivity (Brodkin 2007). Each cage housed one dam and her litter, with *ad libitum* access to autoclaved food and drinking water.

All animal care and experimental procedures were conducted at Comparative Medicine Biomedicum, Karolinska Institutet, Sweden. All procedures were approved by the Ethics Committee on Animal Research, Stockholm North (Dnr 12 837‐2020), and complied with the European Communities Council Directive 2010/63/EU. No exclusion criteria were pre‐specified, and no animals were excluded or died during the study. Detailed information on experimental resources, including catalog numbers and RRIDs, is provided in Table S1.

### 2.2 Probiotic Treatment

The probiotic formulation *Limosilactobacillus reuteri* W192 (*L. reuteri*; batch number: 20G0138), formerly known as *Lactobacillus reuteri* , was provided in a carrier matrix consisting of rice starch and maltodextrin. Both the single‐strain probiotic and the control formulation (carrier matrix only) were supplied by Winclove Probiotics B.V., Amsterdam, the Netherlands. Oral administration via drinking water was chosen over oral gavage to minimize stress in the mice. Pregnant dams were randomly assigned by the experimenter to receive either *L. reuteri* (8 × 10^7^ CFU/mL) or control formulation in their drinking water from GD6 until P7. Animals were allocated to the probiotic or control group without the use of a formal randomization tool. All solutions were freshly prepared each day in sterile drinking water. Water intake was visually assessed daily by the technician, and no differences were noted between groups.

### 2.3 Experimental Design

An overview of the experimental design and timeline is provided in Figure S1. The study was designed in accordance with the 3Rs (Replacement, Reduction, Refinement) to ensure optimal animal welfare. The date of birth was designated as P0. Offspring were weaned at P21 and housed in same‐sex, same‐treatment groups (four animals per cage). Behavioral testing was conducted in adulthood (10–11 weeks of age). A total of 32 mice (*n* = 8 per group: Control Female, Probiotic Female, Control Male, Probiotic Male) were randomly selected, with no dam contributing more than two pups to any one group. However, no formal randomization method (e.g., computer‐generated or algorithm‐based assignment) was used to allocate animals to experimental groups. Tissue analysis was conducted on a random subset of animals (*n* = 6 per group). This sample size was based on prior studies with comparable designs (Siegler Lathrop et al. 2024) and was sufficient to detect group differences in both behavioral and molecular outcomes with established statistical power. The study design adhered to the 3R principles by minimizing the number of animals used while maintaining scientific rigor.

### 2.4 Behavioral Testing

Behavioral testing was conducted between 09:00 and 17:00 under low illumination (40 lux) to minimize stress. Equipment was sanitized before and between sessions. Animals were transported in their home cages to the experimental room and allowed to habituate for at least 30 min to acclimate to the new environment. All animals were naïve to the procedures. Adult female offspring (10 weeks old) were tested first, followed by male adult offspring (11 weeks old) the following week. The tests were conducted in the following order: Light–Dark Box (LDB) test, Three‐Chamber Social Approach Task (3‐CST), and Elevated Plus Maze (EPM), with a one‐day rest period between each test. Behavioral testing was conducted by two researchers, including one who was blinded to group assignments until after statistical analyses were completed. Behavioral and molecular analyses were performed by three different individuals to reduce bias. However, statistical analyses of both behavioral and gene expression data were conducted without blinding.

#### 2.4.1 Light–Dark Box (LDB) Test

The LDB consisted of two equally sized (24 cm × 24 cm) Plexiglas chambers. One chamber was transparent and illuminated by an LED light strip (1600 lux), while the other remained opaque and dark. The two compartments were connected by a rectangular door located in the center wall. Mice were individually placed in the dark compartment at the beginning of the test and allowed to explore the apparatus freely for 5 min. The following behavioral parameters were recorded for each compartment: time spent (in seconds), distance traveled (in centimeters, cm), and the number of rears. Data collection was automated using the Acti‐Mot detection system (TSE Systems, Bad Homburg, Germany), which employs photobeam sensors to track the animals' movements, measuring time spent, distance traveled, and the number of rears in each compartment.

#### 2.4.2 Elevated Plus Maze (EPM) Test

The test mouse was placed at the center of an EPM apparatus, oriented to face one of the open arms, and allowed to explore the maze for 5 min. The EPM apparatus (Kinder Scientific) was constructed from black Plexiglas and comprised two open arms (36 × 5 cm), two enclosed arms (36 × 5 cm), and a central platform (5 × 5 cm), elevated 64 cm above the floor. The walls of the enclosed arms were 16 cm high and made from the same material. The following behavioral parameters were recorded using infrared photo‐beams: the number of entries, time spent (in seconds), and distance traveled in the open arms, closed arms, and intersection (central area) of the maze (in cm).

#### 2.4.3 Three‐Chamber Social Approach Task (3‐CST)

Mice were tested in a three‐chambered Plexiglas box (43.7 × 20 × 35 cm). The test consisted of three 10‐min phases: habituation, sociability, and social novelty. During habituation, the test mouse freely explored the empty apparatus. In the sociability phase, a C57BL/6JRj juvenile mouse (1 month old, same sex), habituated to a grid enclosure, was placed in one of the side chambers, while an identical empty enclosure was placed in the opposite side chamber, allowing the test mouse to explore all three chambers. In the social novelty phase, the familiar conspecific remained, while a new C57BL/6JRj juvenile mouse (1 month old, same sex) was introduced into the previously empty enclosure. Total time in chambers and interaction time (sniffing and physical contact, in seconds) were recorded using an automated video‐tracking system (EthoVision XT, version 11, Noldus, Wageningen, The Netherlands).

### 2.5 RNA Extraction and Quantitative RT‐PCR

Three days after the completion of behavioral testing, mice were euthanized by cervical dislocation between 09:00 and 11:00. The brain, colon, and fecal content from the cecum were rapidly dissected on ice, immediately frozen on dry ice, and stored at −80°C until further analysis. RNA was extracted from the prefrontal cortex (PFC), striatum, and colon using the QIAGEN RNeasy Mini Kit, following the manufacturer's instructions (Cat. No. 74104, Qiagen). Tissue samples were mechanically lysed using a bead‐based homogenization method with 5‐mm stainless steel beads in a TissueLyser II (Qiagen). RNA concentrations were measured using a NanoDrop 2000C spectrophotometer (Thermo Fisher) for quality control. Samples were stored at −80°C until further processing. cDNA was synthesized from purified RNA using the iScript cDNA Synthesis Kit (Bio‐Rad) and stored at −20°C until further use.

Quantitative PCR assays were performed on the QuantStudio 7 Real‐Time PCR System (Applied Biosystems, Life Technologies) using SYBR Green chemistry. The primers used for the assays are listed in Table S2. Peptidylprolyl isomerase A (*Ppia*), hypoxanthine‐guanine phosphoribosyltransferase (*Hprt*), and glyceraldehyde‐3‐phosphate dehydrogenase (*Gapdh*) were selected as housekeeping genes after evaluating multiple candidates highly expressed in the brain regions of interest (Svingen et al. 2015) and identifying those with stable expression across all experimental groups.

### 2.6 16S rRNA Gene Sequencing and Analysis

Cecal content samples were harvested directly from the cecum during dissection and immediately frozen on dry ice. DNA was extracted from cecal microbiota samples using the QIAamp PowerFecal Pro DNA Kit (Qiagen AB, Sweden). Mechanical lysis was performed with 0.5‐mm zirconium beads in a Precellys Evolution Touch homogenizer (Bertin Technologies). The V3–V4 hypervariable region of the bacterial 16S rRNA gene was amplified and sequenced using the Illumina MiSeq platform. Sequencing and initial data processing were performed by Novogene (UK). Further analyses and figure generation were conducted using R (version 4.1.2) and GraphPad Prism 10.

### 2.7 Statistical Analysis

Data were analyzed using GraphPad Prism 10 and R version 4.1.2. Outliers were identified using Grubbs' test (α = 0.05) and excluded if detected. All exclusions are reported in the corresponding figure legends. Normality was assessed using the Shapiro–Wilk test. Results are presented as mean ± standard error of the mean (SEM). For behavioral data (LDB, EPM, and 3‐CST), two‐tailed, two‐way ANOVAs (Treatment × Condition) were conducted separately for males and females. Gene expression data were analyzed using the same approach, with separate two‐way ANOVAs (Treatment × Gene) performed for each sex. When significant main effects or interactions were observed, Bonferroni's post hoc test was used for multiple comparisons. As body weight data were not normally distributed in some groups, comparisons between control and probiotic groups were made using the Mann–Whitney U test. Litter effects were not statistically modeled, as no more than two pups per experimental group were selected from each litter. For microbiome analyses, multiple testing correction was performed using the Benjamini–Hochberg procedure, with a false discovery rate (FDR) threshold of 0.1. Statistical significance was defined as *p* < 0.05. Beta diversity was assessed using Bray–Curtis dissimilarity and visualized via Principal Coordinate Analysis (PCoA), with statistical differences determined by PERMANOVA (adonis2 function, vegan package) using 1000 permutations.

## 3 Results

### 3.1 Long‐Term Effects of Perinatal Exposure to *L. reuteri* on Body Weight

The body weight of male and female offspring perinatally exposed to probiotics, as well as their respective control groups, was evaluated at 3 and 10 weeks of age (Figure S2). Perinatally probiotic‐exposed males exhibited a modest but statistically significant increase in body weight at both 3 and 10 weeks of age (Mann–Whitney *U* test: *U = 10, p* = 0.0079 and *U* = 13, *p* = 0.0493, respectively; Figure S2a,b). In contrast, no significant effects on body weight were observed in female offspring (*p* > 0.1; Figure S2a,b).

### 3.2 Long‐Term Effects of Perinatal Exposure to *L. reuteri* on Anxiety‐Like Behavior

**FIGURE 1:**
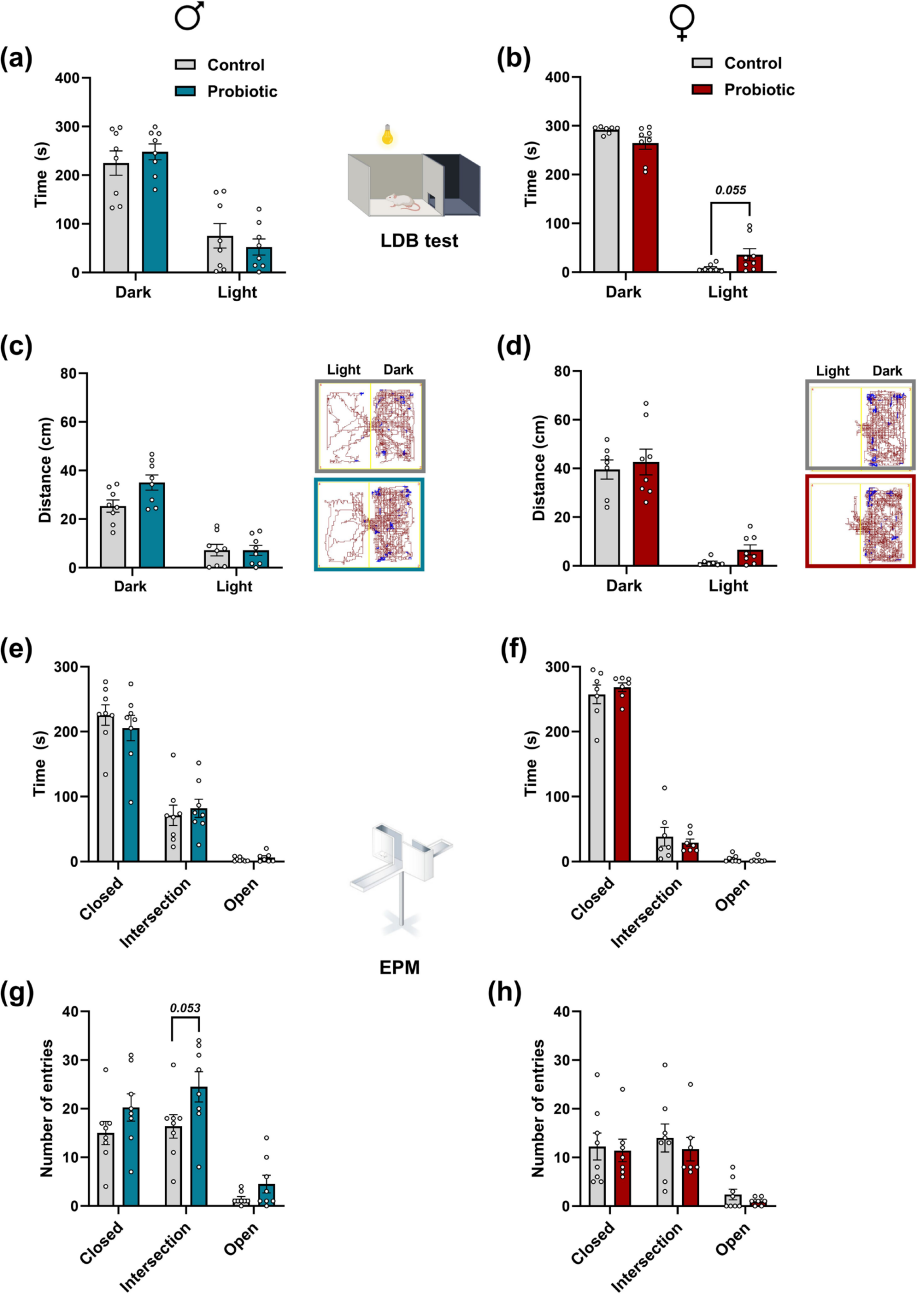
Impact of perinatal exposure to *L. reuteri* on anxiety‐like behavior in adult male and female offspring. (a, b) Time (in seconds) spent in the light and dark compartments during a 5‐min Light–Dark Box (LDB) test for male and female offspring, respectively. (c, d) Distance traveled (in centimeters) in each compartment during the LDB test, along with representative movement patterns. (e, f) Time (in seconds) spent in different zones of the Elevated Plus Maze (EPM) by male and female offspring. (g, h) Number of entries into each zone of the EPM by male and female offspring. All data are presented as mean ± SEM, with adjusted *p*‐values shown after correction for multiple comparisons. *N* = 8 per group, except for probiotic females in the EPM (f, h), where *n* = 7 due to exclusion of one outlier identified by Grubbs' test.

Anxiety‐like behavior was assessed using the LDB and EPM tests. In males, there were no significant treatment or interaction effects for time spent in the dark versus light compartment of the LDB test (*p* > 0.1; Figure 1a). However, for distance traveled, two‐way ANOVA revealed a non‐significant trend toward a treatment effect [*F* (1,28) = 3.535, *p* = 0.0705] and a treatment × compartment interaction [*F* (1,28) = 3.647, *p* = 0.0665]. Although these trends approached statistical significance, they did not meet the predefined threshold (*p* < 0.05), and no post hoc comparisons were conducted.

In females, two‐way ANOVA revealed a significant treatment × compartment interaction for time spent in the light versus dark compartments [F (1,26) = 8.079, *p* = 0.0086]. Although perinatally probiotic‐exposed females tended to spend more time in the light compartment and less time in the dark compartment, these differences did not remain significant after Bonferroni correction (*p* = 0.055; Figure 1b). No significant treatment effects were observed for distance traveled (*p* > 0.1).

In the EPM, there were no significant differences in time spent in the different zones for either males (*p* > 0.1; Figure 1e) or females (*p* > 0.1; Figure 1f). However, for the number of entries, two‐way ANOVA revealed a significant treatment effect [F (1,42) = 8.235, *p* = 0.0064], with perinatally probiotic‐exposed males showing a tendency toward a higher number of entries into the center zone (*p* = 0.053; Figure 1g). No significant differences were found in female offspring regarding the number of entries (*p* > 0.1; Figure 1h).

### 3.3 Sex‐Dependent Long‐Term Effects of Perinatal Exposure to *L. reuteri* on Social Behavior

**FIGURE 2:**
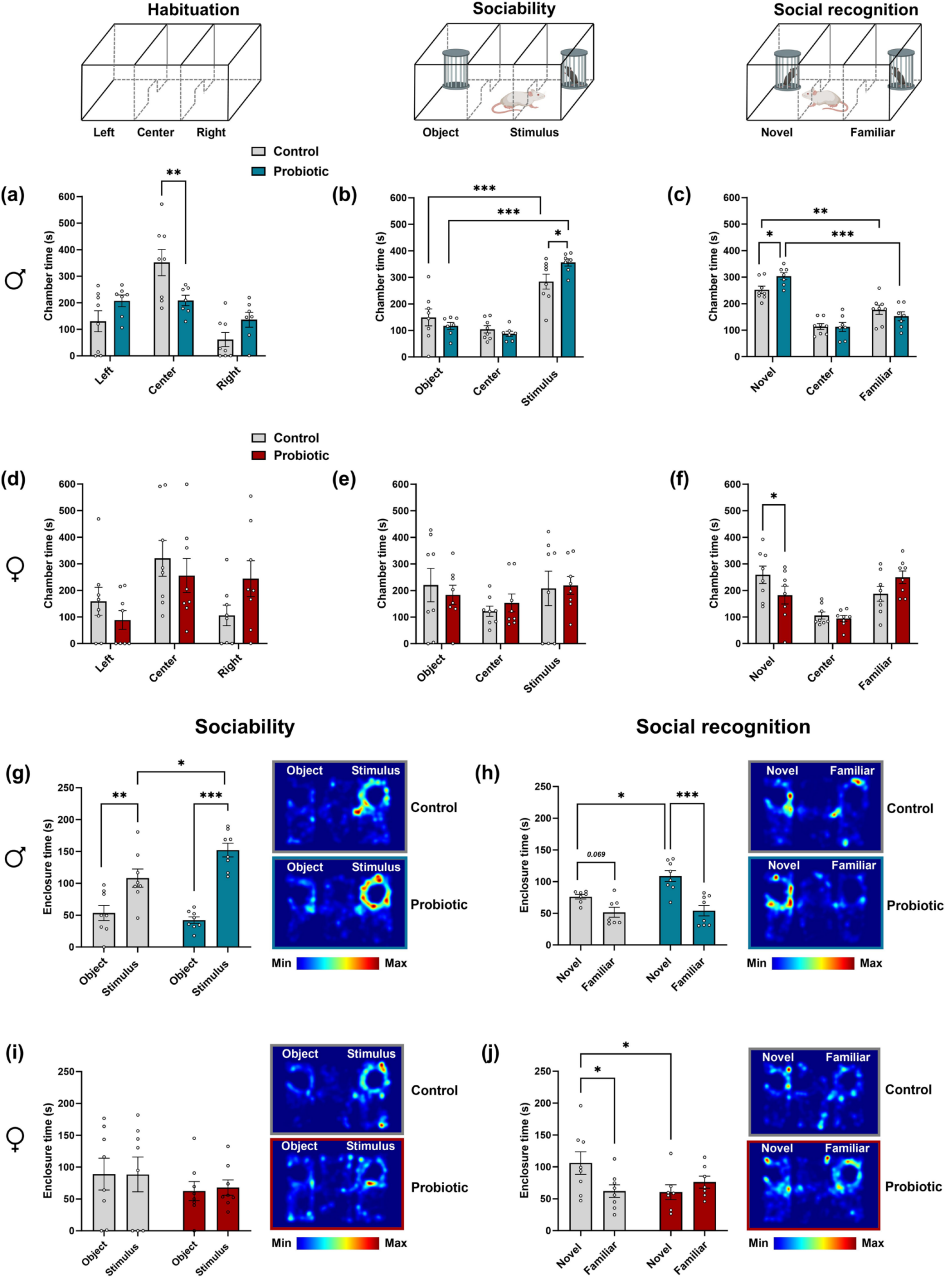
Impact of perinatal exposure to *L. reuteri* on social behavior in adult male and female offspring. (a, d) Time (in seconds) spent in the left, center, and right chambers during the 10‐min habituation phase of the Three‐Chamber Social Approach Task (3‐CST) for male and female offspring, respectively. (b, e) Time (in seconds) spent in the center or side chambers containing either an object or a stimulus mouse during the 10‐min sociability phase of the 3‐CST for male and female offspring, respectively. (c, f) Time (in seconds) spent in the center or side chambers with either a novel stimulus or a familiar mouse during the 10‐min social recognition phase of the 3‐CST for male and female offspring, respectively. (g, i) Time (in seconds) spent in close interaction with either the object or the stimulus mouse during the sociability phase, along with representative heatmaps. (h, j) Time (in seconds) spent in close interaction with either a new stimulus or a familiar mouse during the social recognition phase, along with representative heatmaps. All data are presented as mean ± SEM (*n* = 7–8 per group), with adjusted *p*‐values shown after correction for multiple comparisons. *N* = 8 per group per sex, except for the male probiotic group in (a–c) and the female probiotic group in (i) and (j), where *n* = 7 due to exclusion of one outlier identified by Grubbs' test. *p < 0.05; **p < 0.001; ***p < 0.0001 compared to the respective control group.

Sociability and social recognition were assessed in the 3‐CST. During the habituation phase, a two‐way ANOVA revealed a significant treatment × chamber interaction effect [F (2, 39) = 6.966, *p* = 0.0026], with perinatally probiotic‐exposed males spending significantly less time in the center chamber (*p* = 0.0049). Unlike the control group, which spent most of its time in the center chamber (*p* < 0.0001), perinatally probiotic‐exposed males spent a similar amount of time in all chambers (Figure 2a). No significant differences were found in perinatally probiotic‐exposed females (*p* > 0.1; Figure 2d).

During the sociability phase, two‐way ANOVA revealed a significant treatment × chamber interaction effect [F (2,39) = 3.538, *p* = 0.0387], with perinatally probiotic‐exposed males spending significantly more time in the chamber containing the stimulus mouse compared to their respective control group (*p* = 0.021; Figure 2b). Further analysis [ANOVA: treatment × condition (stimulus vs. object): F (1,28) = 6.239, *p* = 0.0186] showed that perinatally probiotic‐exposed males spent significantly more time in close interaction with the stimulus mouse than their control group (*p* = 0.0179; Figure 2g). In contrast, no significant differences were found in perinatally probiotic‐exposed females for either chamber time or time spent in close interaction with the stimulus mouse (*p* > 0.1; Figure 2e,i).

During the social recognition phase, two‐way ANOVA revealed a significant treatment × chamber interaction effect [F (2,39) = 3.364, *p* = 0.0449], with perinatally probiotic‐exposed males spending a significantly greater amount of time in the chamber containing the novel stimulus mouse compared to their control group (*p* = 0.02; Figure 2c). Further analysis [ANOVA: treatment effect: F (1,26) = 5.410, *p* = 0.0281] showed that perinatally probiotic‐exposed males spent significantly more time in close interaction with the novel mouse than their control group (*p* = 0.0105). Notably, perinatally probiotic‐exposed males showed a strong preference for the novel stimulus mouse over the familiar one (*p* < 0.0001; Figure 2h), while the control group showed a weaker, non‐significant preference (*p* = 0.069; Figure 2h).

For female offspring, two‐way ANOVA revealed a significant treatment × chamber interaction effect [F (2,42) = 3.785, *p* = 0.0308], with perinatally probiotic‐exposed females spending significantly less time in the chamber containing the novel stimulus mouse compared to their control group (*p* = 0.038; Figure 2f). Further analysis [ANOVA: treatment effect: F (1,26) = 5.320, *p* = 0.0293] revealed that perinatally probiotic‐exposed females also spent significantly less time in close interaction with the novel stimulus mouse compared to their control group (*p* = 0.0388). In contrast to the control group, which showed a significant preference for the novel stimulus over the familiar one (*p* = 0.0392; Figure 2j), perinatally probiotic‐exposed females did not show any preference (*p* > 0.1; Figure 2j).

### 3.4 Perinatal *L. reuteri* Exposure Alters Key Gene Expression in the PFC of Adult Offspring

Building on our previous research and current literature, we evaluated the expression of key genes in the prefrontal cortex (PFC) associated with distinct neurobiological processes. These included genes involved in sociability and social cognition—*Avpr1a* (arginine vasopressin receptor 1A), *Avpr1b* (arginine vasopressin receptor 1B), and *Oxtr* (oxytocin receptor); genes related to the transport of small soluble bacterial components such as peptidoglycans—*Slc15a1* (PepT1), Slc15a2 (PepT2), Slc46a2, and Slc46a3; genes involved in synaptic and myelin integrity—Bdnf (brain‐derived neurotrophic factor), *Ppp1r1b* (dopamine‐ and cAMP‐regulated phosphoprotein of 32 kDa; DARPP‐32), *Syp* (synaptophysin), *Mag* (myelin‐associated glycoprotein), *Mbp* (myelin basic protein), and *Mog* (myelin oligodendrocyte glycoprotein); and genes associated with microglial activation and immune signaling—*Cx3cr1* (CX3C motif chemokine receptor 1), *Itgam* (cluster of differentiation 11b; CD11b), Aif1 (ionized calcium‐binding adaptor molecule 1; Iba‐1), *Il10* (interleukin‐10), *Il6* (interleukin‐6), and *Trem2* (triggering receptor expressed on myeloid cells 2).

**FIGURE 3:**
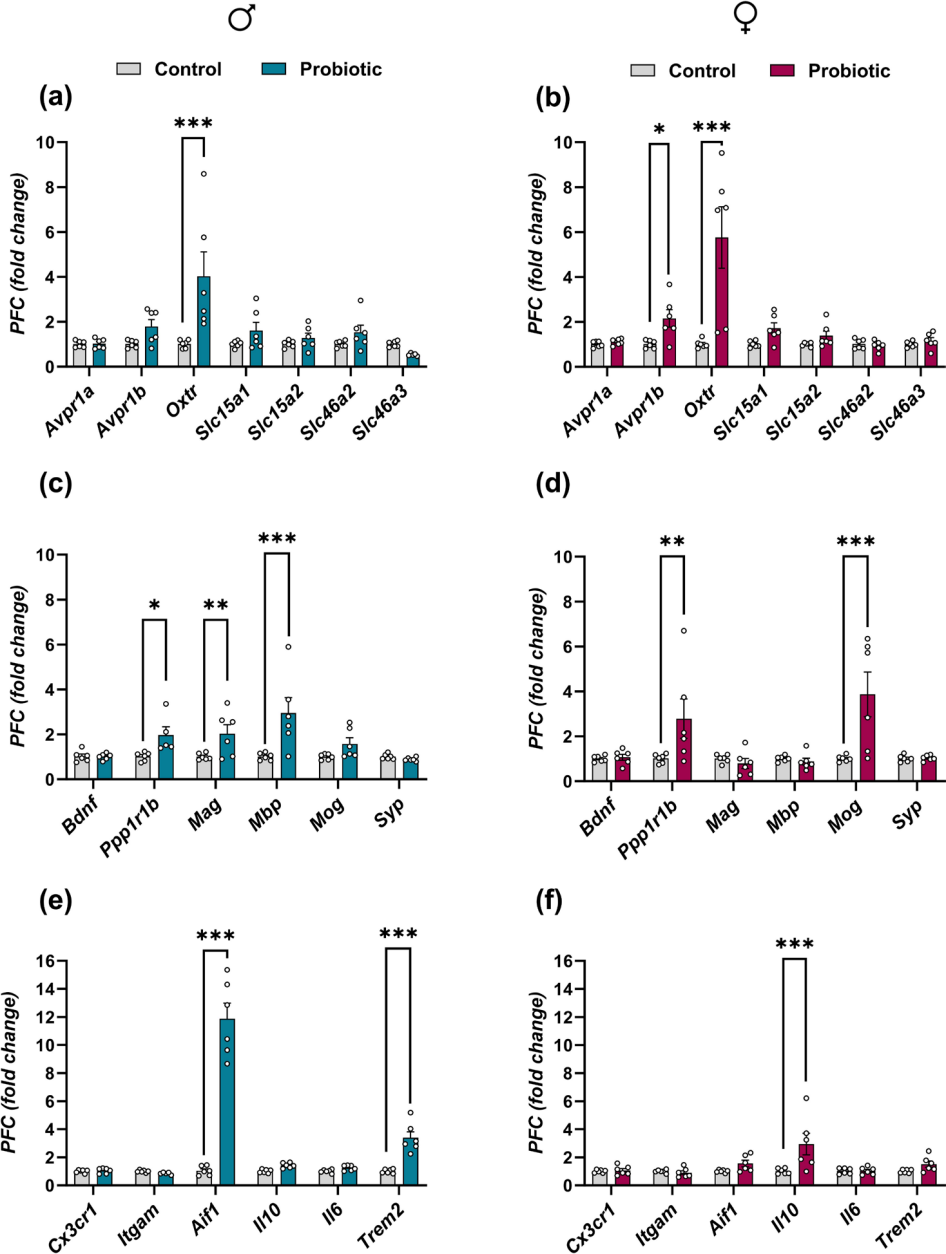
Impact of perinatal exposure to *L. reuteri* on the expression of key genes in the prefrontal cortex (PFC) of male and female offspring. (a–f) Expression of genes in the PFC of male and female offspring. (a, b) Neuropeptide receptor genes associated with social behavior, along with bacterial peptidoglycan transporter‐related genes, in male and female offspring, respectively. (c, d) Genes involved in synaptic function and myelin integrity in male and female offspring, respectively. (e, f) Genes related to microglial function and neuroimmune responses in male and female offspring, respectively. All data are presented as mean ± SEM, with adjusted *p*‐values shown after correction for multiple comparisons. *N* = 6 per group for all genes and both sexes, except for *Ppp1r1b* (DARPP‐32) in the male probiotic group (c), where *n* = 5 due to exclusion of one outlier identified by Grubbs' test. *p < 0.05; **p < 0.001; ***p < 0.0001 compared to the respective control group. Gene abbreviations are defined in the main text.

Gene expression analysis of neuropeptide receptors and PGN transporters in the PFC revealed differential effects of perinatal *L. reuteri* exposure in males and females (Figure 3a,b). In males, a two‐way ANOVA indicated significant main effects of treatment [F(1,69) = 13.90, *p* = 0.0004], gene [F(6,69) = 5.407, *p* = 0.0001], and a treatment × gene interaction [F(6,69) = 5.354, *p* = 0.0001]. Bonferroni's post hoc tests identified a robust increase in *Oxtr* expression in perinatally probiotic‐exposed males (*p* < 0.0001; Figure 3a), while expression levels of *Avpr1a*, *Avpr1b*, and the PGN transporters *Slc15a1*, *Slc15a2*, and *Slc46a2* were unchanged (*p* > 0.1). In females, similar main effects were observed for treatment [F(1,70) = 23.35, *p* < 0.0001], gene [F(6,70) = 9.202, *p* < 0.0001], and their interaction [F(6,70) = 9.170, *p* < 0.0001]. Post hoc comparisons showed a marked upregulation of *Oxtr* (*p* < 0.0001; Figure 3b), as well as a modest but significant increase in *Avpr1b* (*p* = 0.0448; Figure 3b), while *Avpr1a* remained unaffected. None of the PGN transporters examined—including *Slc15a1*, *Slc15a2*, and *Slc46a2*—were significantly altered in probiotic‐treated females (*p* > 0.1).

Analysis of genes related to synaptic function and myelin integrity in the male PFC revealed significant effects of perinatal *L. reuteri* exposure. A two‐way ANOVA showed main effects of treatment [F(1,59) = 22.21, *p* < 0.0001], gene [F(5,59) = 4.104, *p* = 0.0029], and a treatment × gene interaction [F(5,59) = 4.123, *p* = 0.0028]. Bonferroni's post hoc test indicated significantly elevated expression of *Ppp1r1b* (*DARPP‐32; p* = 0.0187), *Mag (p* = 0.0083), and *Mbp* (*p* < 0.0001) in perinatally probiotic‐treated males (Figure 3c). In contrast, expression of *Bdnf*, *Mog*, and *Syp* did not differ significantly between groups. In the PFC of female offspring, two‐way ANOVA revealed significant main effects of treatment [F(1,60) = 10.18, *p* = 0.0023], gene [F(5,60) = 5.263, *p* = 0.0005], and their interaction [F(5,60) = 5.249, *p* = 0.0005]. Bonferroni's post hoc analysis showed a significant increase in *Ppp1r1b* (DARPP‐32; *p =* 0.0025) and *Mog* (*p* < 0.0001) expression in perinatally probiotic‐exposed females (Figure 3d). No significant differences were found in the expression of *Bdnf*, *Mag*, *Mbp*, or *Syp* (*p* > 0.1 for all).

Expression analysis of immune‐ and microglia‐related genes in the male PFC revealed robust changes following perinatal *L. reuteri* exposure. A two‐way ANOVA showed highly significant effects of treatment [F(1,59) = 126.4, *p* < 0.0001], gene [F(5,59) = 75.40, *p* < 0.0001], and their interaction [F(5,59) = 74.37, *p* < 0.0001]. Bonferroni's post hoc analysis identified strong upregulation of *Aif1* (Iba‐1) and *Trem2* in the probiotic group (*p* < 0.0001 for both; Figure 3e). In contrast, no significant changes were found in *Cx3cr1*, *Itgam* (CD11b), *Il6*, or *Il10* (*p* > 0.1 for all). In the female PFC, a two‐way ANOVA revealed significant main effects of treatment [F(1,60) = 11.43, *p* = 0.0013], gene [F(5,60) = 4.430, *p* = 0.0017], and their interaction [F(5,60) = 4.395, *p* = 0.0018]. Among the genes analyzed, only *Il10* showed a significant increase in expression following probiotic exposure, as confirmed by Bonferroni's post hoc test (*p* < 0.0001; Figure 3f). No significant differences were observed for *Cx3cr1*, *Itgam*, *Aif1, Trem2*, or *Il6* (*p* > 0.1).

### 3.5 Perinatal *L. reuteri* Exposure Alters Key Gene Expression in the Striatum of Adult Offspring

**FIGURE 4:**
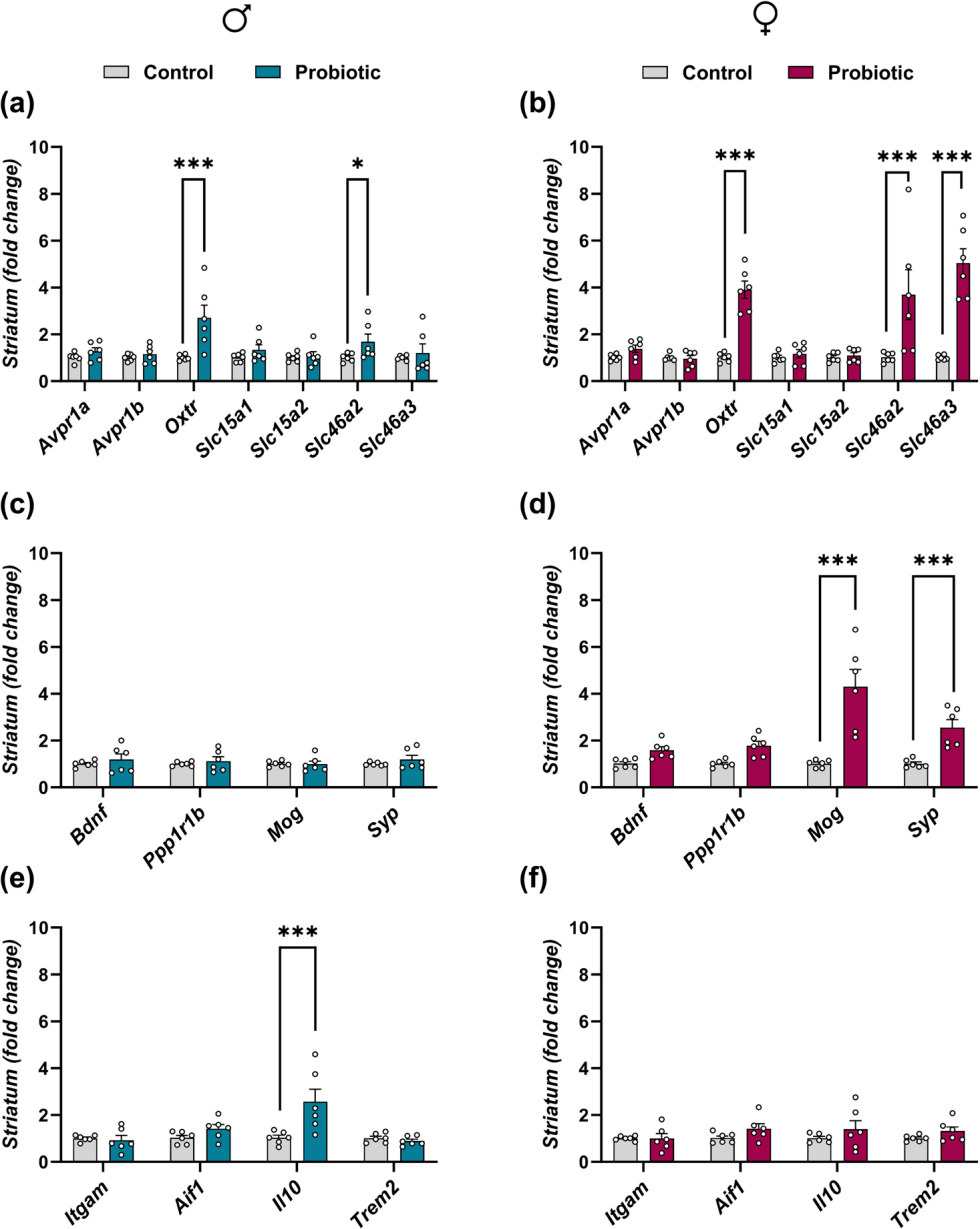
Impact of perinatal exposure to *L. reuteri* on the expression of key genes in the striatum of male and female offspring. (a–f) Expression of genes in the striatum of male and female offspring. (a, b) Neuropeptide receptor genes associated with social behavior, along with bacterial peptidoglycan transporter‐related genes, in male and female offspring, respectively. (c, d) Genes involved in synaptic function and myelin integrity in male and female offspring, respectively. (e, f) Genes related to microglial function and neuroimmune responses in male and female offspring, respectively. All data are presented as mean ± SEM (*n* = 6 per group), with adjusted *p*‐values after correction for multiple comparisons. *p < 0.05; ***p < 0.0001 compared to the respective control group. Gene abbreviations are defined in the main text.

Gene expression analysis of neuropeptide receptors and PGN transporters in the male striatum revealed significant effects of perinatal *L. reuteri* exposure. In male offspring, a two‐way ANOVA indicated significant main effects of treatment [F(1,70) = 15.89, *p* = 0.0002], gene [F(6,70) = 3.196, *p* = 0.0079], and a treatment × gene interaction [F(6,70) = 3.248, *p* = 0.0071]. Bonferroni's post hoc analysis showed that *Oxtr* expression was significantly increased in perinatally probiotic‐exposed males (*p* < 0.0001; Figure 4a), along with a modest but significant increase in *Slc46a2* (*p* = 0.0390; Figure 4a). In contrast, *Avpr1a*, *Avpr1b*, *Slc15a1* (PepT1), *Slc15a2* (PepT2), and *Slc46a3* were unchanged (*p* > 0.1 for all; Figure 4a). In female offspring, significant effects were also observed for treatment [F(1,70) = 58.56, *p* < 0.0001], gene[F(6,70) = 11.40, *p* < 0.0001], and their interaction [F(6,70) = 11.43, *p* < 0.0001]. A robust upregulation of *Oxtr*, *Slc46a2*, and *Slc46a3* was found in perinatally probiotic‐exposed females (*p* < 0.0001 for all; Figure 4b), while no significant changes were found for *Avpr1a*, *Avpr1b*, *Slc15a1*, or *Slc15a2* (*p* > 0.1 for all; Figure 4b).

Analysis of synaptic‐ and myelin‐related gene expression in the male striatum did not reveal any main effects of treatment [F(1,40) = 1.552, *p* = 0.22], gene [F(3,40) = 0.2326, *p* = 0.8731], or treatment × gene interaction [F(3,40) = 0.2449, *p* = 0.8645]; therefore, no post hoc comparisons were performed (Figure 4c). In contrast, female offspring exhibited robust changes in striatal expression of these genes. A two‐way ANOVA showed significant main effects of treatment [F(1,40) = 52.54, *p* < 0.0001], gene [F(3,40) = 8.351, *p* = 0.0002], and their interaction [F(3,40) = 8.401, *p* = 0.0002]. Bonferroni's post hoc analysis revealed that *Mog* and *Syp* were significantly upregulated in the probiotic group (*p* < 0.0001 and *p* = 0.0008, respectively; Figure 4d). No other significant differences were observed.

Analysis of microglial and immune‐related gene expression in the male striatum revealed significant main effects of treatment [F(1,40) = 7.356, *p* = 0.0098], gene [F(3,40) = 6.200, *p* = 0.0015], and their interaction [F(3,40) = 5.890, *p* = 0.0020]. Bonferroni's post hoc test identified a significant increase in *Il10* expression in probiotic‐exposed males (*p* < 0.0001; Figure 4e), while no significant differences were observed for *Itgam* (CD11b), *Aif1* (Iba‐1), or *Trem2* (*p* > 0.1 for all). In female offspring, two‐way ANOVA revealed a significant main effect of treatment [F(1,40) = 4.345, *p* = 0.0436], but no significant effects of gene or treatment × gene interaction (*p* > 0.1 for both). Post hoc analysis confirmed that no individual genes were significantly altered (Figure 4f).

### 3.6 Perinatal *L. reuteri* Increases *Il10* Gene Expression in the Colon of Adult Males

**FIGURE 5:**
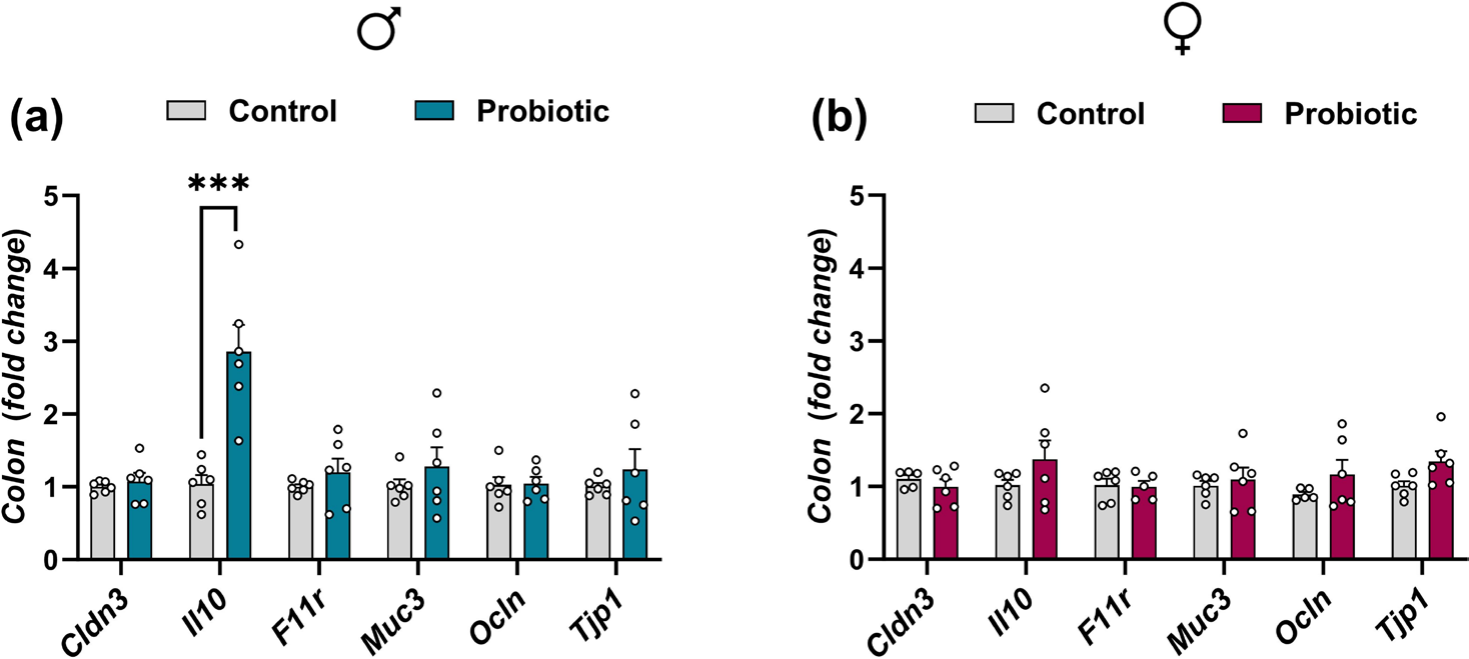
Impact of perinatal exposure to *L. reuteri* on the expression of tight junction, mucosal, and immune‐related genes in the colons of male and female offspring. (a) Male and (b) female offspring. All data are presented as mean ± SEM, with adjusted *p*‐values shown after correction for multiple comparisons. *n* = 6 per group for all genes, except for *F11r* (JAM‐A) in the probiotic female group (b), where *n* = 5 due to exclusion of outliers identified using Grubbs' test. ***p < 0.0001 compared to the respective control group. Gene abbreviations are defined in the main text.

Colon gene expression analysis revealed sex‐specific effects of perinatal *L. reuteri* exposure. In male offspring, a two‐way ANOVA showed significant main effects of treatment [F(1,60) = 17.68, *p* < 0.0001], gene [F(5,60) = 7.843, *p* < 0.0001], and their interaction [F(5,60) = 7.360, *p* < 0.0001]. Bonferroni's post hoc test indicated a marked increase in *Il10* expression in perinatally probiotic‐exposed males (*p* < 0.0001; Figure 5a). No significant differences were observed in the expression of *Cldn3* (claudin‐3), *F11r* (junctional adhesion molecule A; JAM‐A), *Muc3* (mucin‐3), or *Ocln* (occludin) (*p* > 0.1 for all). In female offspring, a two‐way ANOVA revealed no significant main effect of treatment or treatment × gene interaction (*p* > 0.1 for all), no post hoc comparisons were performed (Figure 5b).

### 3.7 Long‐Term Effects of Perinatal *L. reuteri* on Gut Microbiota Composition of Adult Offspring

**FIGURE 6:**
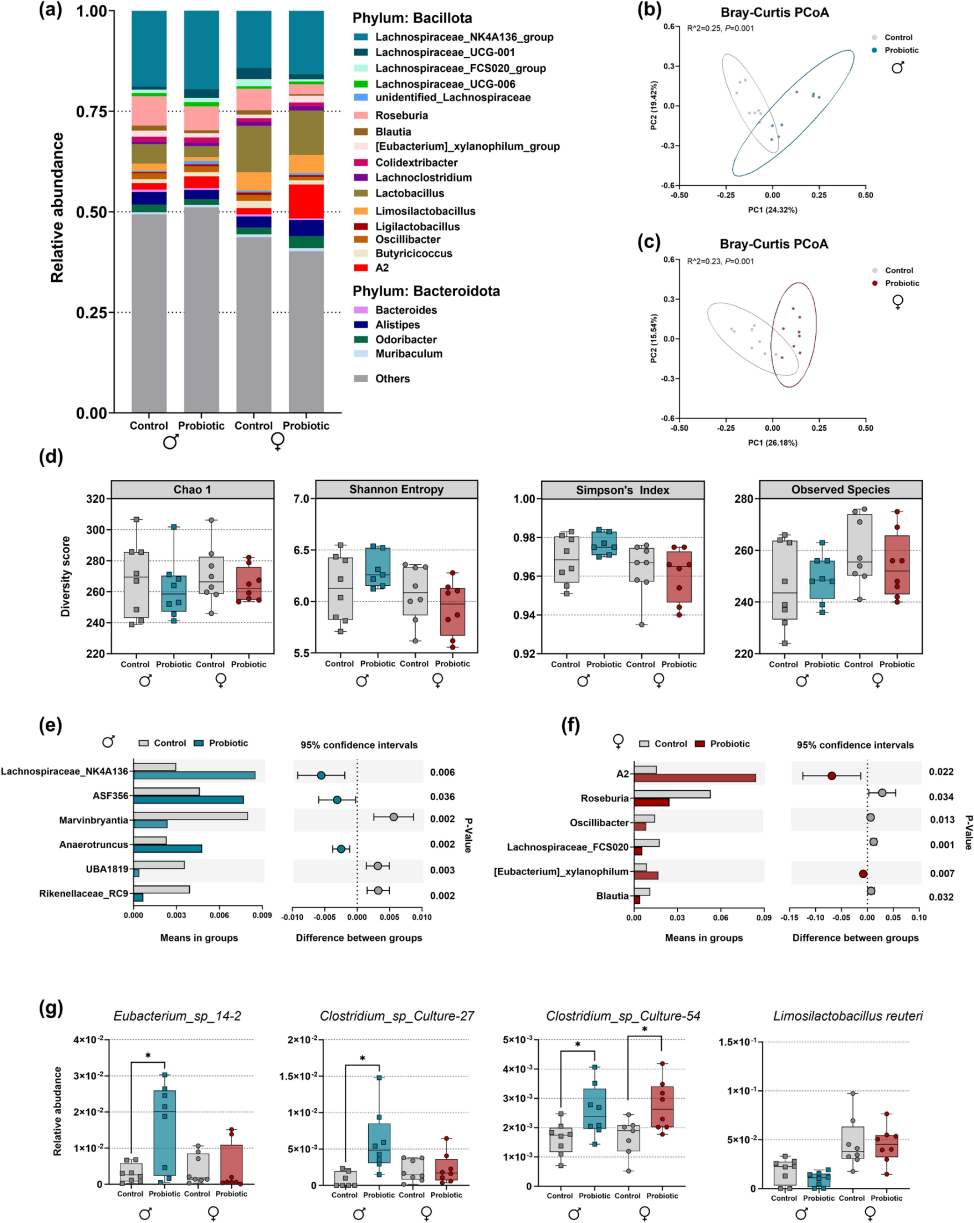
Long‐lasting changes in cecal microbiota composition of perinatally probiotic‐exposed male and female offspring. (a) Relative abundance of the top 20 taxa at the genus level. (b, c) Principal Coordinates Analysis (PCoA) of beta diversity in male and female offspring. (d) Alpha diversity in male and female offspring, assessed using the Chao1, Shannon Entropy, Simpson's Index, and Observed Species indices. (e, f) Relative abundance and 95% confidence intervals of bacterial genera showing significant changes in perinatally probiotic‐exposed male (e) and female (f) offspring compared to their respective controls. (g) Relative abundance of bacterial species showing changes in perinatally probiotic‐exposed male and female offspring compared to their respective controls. *p < 0.05 compared to the respective control group. *N* = 8 per group.

The relative abundance of the top 20 taxa at the genus level for perinatally probiotic‐exposed male and female offspring, as well as their respective control groups, is shown in Figure 6a. Principal coordinates analysis (PCoA) based on the Bray‐Curtis distance matrix identified significant differences in beta‐diversity in both sexes, indicating variations in the overall diversity (PERMANOVA: Males: *R* ^2^ = 0.25, *p* = 0.001; Females: *R* ^2^ = 0.23, *p* = 0.001; Figure 6b,c). However, there were no significant differences in various parameters of alpha‐diversity (Chao 1, Simpson's index, and Shannon Entropy), which describe community complexity in terms of species richness and evenness, when analyzed at the genus level (Figure 6d).

We found that the relative abundance of several genera within the Firmicutes phylum, specifically from the Lachnospiraceae family, including *Lachnospiraceae_NK4A136*, *ASF356*, and *Anaerotruncus*, were significantly increased in the perinatally probiotic‐exposed male offspring (Figure 6e). In contrast, *Marvinbryantia*, *UBA1819*, and *Rikenellaceae* from the Bacteroidota phylum, which are typically Gram‐negative, were significantly decreased (Figure 6e). On the other hand, *A2* and *Eubacterium_xylanophilum_group* were significantly increased in the prenatally probiotic‐exposed female offspring, while *Roseburia*, *Oscillibacter*, *Lachnospiraceae_FCS020*, and *Blautia* showed a significant decrease in abundance (Figure 6f).

At the species level, the relative abundance of Gram‐positive bacteria, including *Eubacterium_sp_14‐2*, *Clostridium_sp_Culture‐27*, and *Clostridium_sp_Culture‐54*, was significantly increased in perinatally probiotic‐exposed male offspring, as well as *Clostridium_sp_Culture‐54* in exposed females (Figure 6g). In contrast, the Gram‐positive species *Lachnospiraceae_bacterium_28‐4* significantly decreased in perinatally probiotic‐exposed female offspring (data not shown). Interestingly, we found that adult females harbor more *L. reuteri* than males but did not observe any long‐term impact of perinatal exposure to *L. reuteri* in either sex (Figure 6g).

## 4 Discussion

Emerging evidence underscores the pivotal role of the maternal gut microbiota during pregnancy and lactation in shaping early‐life neural circuits and influencing long‐term developmental outcomes in offspring. These insights have spurred interest in targeting the maternal microbiota to promote healthy neurodevelopment. In this study, we show that perinatal supplementation with the probiotic *L. reuteri* , administered from GD6 to P7, significantly alters long‐term neurodevelopmental trajectories in a sex‐dependent manner. Notably, male—but not female—offspring exposed to perinatal *L. reuteri* exhibited enhanced social motivation and cognition, as evidenced by improved sociability (i.e., the tendency to seek social interaction) and social recognition in the 3‐CST. This male‐specific behavioral phenotype was accompanied by selective upregulation of *Aif1* (Iba1) and *Trem2* in the prefrontal cortex, as well as increased expression of the anti‐inflammatory cytokine *Il10* in both the striatum and colon, suggesting a sex‐dependent immune signature that may support enhanced social functioning in males. Moreover, males also exhibited an increase in the relative abundance of beneficial gut microbial taxa—a change not observed in females—further supporting a microbiota–immune–brain mechanism underlying the observed behavioral improvements. Together, these findings highlight that perinatal probiotic intervention can shape brain and behavioral development through sex‐specific gut–immune–brain pathways, underscoring the need for precision microbiota‐based strategies tailored by sex.

Previous animal studies have demonstrated the beneficial effects of *L. reuteri* treatment in several mouse models of ASD during postnatal life (i.e., starting after weaning), particularly on social behaviors such as sociability, social novelty, and reciprocal social interaction (Buffington et al. 2016; Sgritta et al. 2019). However, these studies examined only male offspring. Interestingly, another study investigating *L. reuteri* treatment in a mouse model of ASD (Shank3 −/− mice) that included both sexes found partially beneficial effects on sociability—but only in males—when assessed using the 3‐CST (Tabouy et al. 2018). Notably, the treatment began at 8 weeks of age, and effects on social recognition were not assessed. In contrast, our study revealed striking, sex‐dependent long‐term effects of perinatal *L. reuteri* supplementation on sociability and social recognition, emphasizing the importance of both the timing of intervention and the sex‐specific sensitivity of social brain networks to microbiota‐based modulation. This is particularly relevant in light of well‐documented sex differences in neurodevelopmental disorders, such as ASD, where males are more frequently diagnosed than females, even as awareness of female presentations continues to grow (Lord et al. 2020). The enhanced social behavior in males may reflect a complex interplay between microbiota modulation, host genetics, and neuroimmune regulation. Specifically, we found consistent upregulation of the anti‐inflammatory cytokine *Il10* in the striatum and colon of perinatally probiotic‐exposed male offspring, suggesting a potential neuroprotective mechanism. The role of IL‐10 in mitigating neuroinflammation is well established (Saraiva et al. 2020), and neuroinflammation has been implicated in the pathophysiology of ASD (Hughes et al. 2023). Together, our findings highlight the perinatal period as a sensitive window during which microbiota‐based interventions—such as *L. reuteri* —can exert enduring, sex‐specific effects on social behavior, potentially mediated by region‐ and sex‐specific upregulation of the anti‐inflammatory cytokine IL‐10 in both brain and gut tissues.

Emerging clinical studies also support the notion that *L. reuteri* exerts beneficial effects on brain social networks. In a recent double‐blind, randomized, placebo‐controlled clinical trial in children with ASD, a combination of *L. reuteri* (ATCC PTA 6475 and DSM 17938) was tested (Mazzone et al. 2024). While the combination did not alter overall autism severity, restricted and repetitive behaviors, microbiome composition, or immune profile, it led to significant improvements in social functioning. In the same study, using the BTBR T+ Itpr3tf/J mouse model of idiopathic ASD, the authors showed that the *L. reuteri* strain ATCC PTA 6475—but not its parental strain ATCC 55730 (from which DSM 17938 is derived)—reversed social deficits without affecting locomotor activity or repetitive behaviors, suggesting potential strain‐specific effects (Mazzone et al. 2024). In a recent phase Ib crossover trial in adolescents and adults with ASD, which tested SB‐121 (a formulation of *L. reuteri* with dextran microparticles and maltose), the authors reported improvements in adaptive behavior and trends toward enhanced social preference (Schmitt et al. 2023). Taken together, these preliminary clinical findings support the notion that *L. reuteri* modulates social brain networks from rodents to humans. Although these studies have focused exclusively on male subjects (mice or individuals with ASD), our findings extend this work by showing that maternal supplementation with *L. reuteri* during gestation and early postnatal life (GD 6 to P3) improves sociability and social recognition in adult male offspring of BALB/c dams. No significant effects were observed in females. This male‐specific effect is consistent with both preclinical and clinical studies and indicates potential sex‐dependent sensitivity to microbiota‐based interventions in ASD‐relevant behaviors.

Interestingly, we observed increased expression of the oxytocin receptor (*Oxtr*) in both PFC and striatum of male and female offspring following perinatal *L. reuteri* supplementation. Oxytocin plays a pivotal role in social bonding and emotional regulation (Froemke and Young 2021), and the upregulation of *Oxtr* gene expression may underlie neurobiological adaptations to probiotic exposure. However, despite this shared *Oxtr* gene upregulation, only males exhibited enhanced social behavior, while females showed impaired social recognition. Interestingly, probiotic‐exposed females displayed a modest reduction in anxiety‐like behavior in the LDB test, suggesting more context‐dependent or circuit‐specific effects. These sex differences may necessitate tailored microbial interventions—such as multi‐species probiotics or synbiotics—to achieve consistent behavioral outcomes (Chan et al. 2023; Siegler Lathrop et al. 2024). Our findings align with previous work in prairie voles, where female—but not male—voles treated with live *L. reuteri* showed reduced affiliative behavior compared to heat‐killed controls (Donovan et al. 2023), emphasizing the complexity of sex‐dependent behavioral responses to microbial interventions.

Importantly, the enhanced social behavior in males cannot be explained by *Oxtr* gene expression alone. Instead, our data suggest that neuroimmune modulation plays a key role. Specifically, male offspring exhibited a significant increase in expression of the anti‐inflammatory cytokine *Il10* in the striatum and colon, as well as upregulation of microglial markers *Aif1* (Iba‐1) and *Trem2* in the PFC—changes not observed in females in these regions. In contrast, female offspring showed a modest increase in Il10 expression in the PFC only. These findings indicate that sex‐specific immune adaptations may underlie the pro‐social effects of early‐life *L. reuteri* exposure. IL‐10 is well known for its role in suppressing neuroinflammation, a process implicated in ASD and related neurodevelopmental disorders (Hughes et al. 2023; Saraiva et al. 2020). The convergent increase in *Il10* gene expression, together with microglial gene activation, supports the notion that immunoregulatory signaling constitutes a critical mechanism linking the early‐life microbiome to long‐term behavioral outcomes in a sex‐dependent manner.

An intriguing observation in our study was the upregulation of genes critical for myelin integrity, such as *Mbp* and *Mog*, following perinatal *L. reuteri* supplementation. Prenatally probiotic‐exposed female offspring showed consistent increases in *Mog* expression in both the prefrontal cortex and striatum, along with elevated levels of *Syp*, a synaptic marker, in the striatum, suggesting region‐specific effects. In contrast, exposed male offspring exhibited changes in *Mbp* expression exclusively in the prefrontal cortex. Growing evidence suggests that oligodendrogenesis and myelination are modulated by the gut microbiota (Sauma and Casaccia 2020). Using GF mice, Hoban et al. (2016) demonstrated that the microbiome is essential for the appropriate and dynamic regulation of myelin‐related genes in the prefrontal cortex. Additionally, recent studies have shown that microbiota‐derived metabolites, such as 4‐ethylphenyl sulfate (4EPS)—elevated in a subset of children with ASD and gastrointestinal disturbances (Needham et al. 2021)—can impair oligodendrocyte maturation (Needham et al. 2022). Interestingly, previous studies have reported a link between sociability and brain size in BALB/c mice (Kim et al. 2012), with smaller brains associated with reduced social behavior. Future studies should explore whether perinatal *L. reuteri* shapes social brain networks—both structurally and functionally—to mediate improved social behavior, and whether these effects differ by sex.

Perinatal exposure to *L. reuteri* had lasting effects on the composition of the gut microbiota in both male and female offspring. Unlike our previous studies using a multispecies probiotic mixture that did not include *L. reuteri* —in which both male and female adult offspring showed an increase in anti‐inflammatory bacteria (Siegler Lathrop et al. 2024)—perinatal *L. reuteri* alone induced more complex and sex‐specific microbial shifts. In male offspring, we observed a significant increase in the relative abundance of *Lachnospiraceae_NK4A136, ASF356*, and *Anaerotruncus*, all genera within the Firmicutes phylum, specifically the Lachnospiraceae family. These taxa are known contributors to short‐chain fatty acid (SCFA) production, particularly butyrate, which has anti‐inflammatory properties and supports gut health and immune function (Vacca et al. 2020; Zhang et al. 2019). Conversely, several members of the Bacteroidota phylum—*Marvinbryantia*, *UBA1819*, and *Rikenellaceae*—were significantly reduced. These taxa are involved in the degradation of complex carbohydrates and dietary fiber (Flint et al. 2012), suggesting a shift in microbial functions related to metabolism and immune regulation. Despite these changes, no detrimental effects on intestinal barrier integrity were observed, as indicated by colon gene expression data. On the contrary, we detected an increase in *IL‐10* expression in the colon of male offspring, although the functional significance remains to be fully elucidated.

In female offspring, there was an increase in specific taxa associated with metabolism (*A2* and *Eubacterium_xylanophilum_group*), but a decrease in several beneficial genera—*Roseburia*, *Oscillibacter*, *Lachnospiraceae_FCS020*, and *Blautia*—commonly associated with butyrate production and gut‐brain health (Singh et al. 2022). These changes may reflect a sex‐specific reshaping of the gut microbiota with potential consequences for immune and neurodevelopmental outcomes. Further research is needed to determine the long‐term functional implications of these shifts, particularly in relation to the behavioral and neurodevelopmental differences observed in our study.

The mechanisms underlying the effects of probiotics during perinatal brain development are still not fully understood. The behavioral improvements in males following perinatal *L. reuteri* supplementation are linked to a coordinated upregulation of genes in the PFC and striatum that govern social behavior, neural plasticity, myelination, and neuroimmune modulation. In contrast, females exhibited gene expression changes with different regional and functional profiles, which may be insufficient to support enhanced social behavior. These findings underscore the region‐ and sex‐specific integration of gut–brain signals during early development. Future research employing integrative approaches–including functional ultrasound imaging of brain networks–will be essential to more precisely link behavioral phenotypes with brain functional connectivity and gene expression changes.

Recent studies have shown that, while the intrauterine environment is typically sterile, maternal microbiota‐derived molecules can influence fetal brain development via the placenta (Pessa‐Morikawa et al. 2022; Vuong et al. 2020). Among these, bacterial cell wall fragments such as peptidoglycans (PGNs) are of particular interest due to their immunogenic properties. We recently demonstrated that PGN fragments, administered to pregnant females via the drinking water, can cross the placenta and reach the fetal brain, where they may modulate neurodevelopmental processes by engaging specific transporters and innate immune receptors (Martinez Sanchez et al. 2025). In our previous study using multispecies probiotic supplementation during pregnancy, we found a persistent upregulation of PGN transporters in the PFC of adult male offspring (Siegler Lathrop et al. 2024). In the present study, we observed a significant increase in the PGN transporters *Slc46a2* and *Slc46a3* in the striatum of females and Slc46a2 in males as well, following perinatal *L. reuteri* exposure. These observations raise the possibility that PGN‐derived fragments from maternal probiotic intake contribute to neurodevelopmental programming in a sex‐dependent manner through altered transporter expression and signaling in the developing brain. Furthermore, others have demonstrated that the anxiolytic action of a multispecies probiotic in BALB/c mice is independent of bacterial viability (Chan et al. 2023), supporting the idea that microbial structural components may be sufficient to confer neurobehavioral effects.

Our findings should be interpreted in light of several limitations. First, although both sexes were consistently included across behavioral, gene expression, and microbiota analyses, we did not monitor the estrous cycle of perinatally probiotic‐exposed adult BALB/c female offspring. This is relevant, as sociability in female mice can vary across estrous stages in the 3‐CST and may be influenced by visual cues from the novel mouse (Chari et al. 2020). However, comparisons with previous work are limited by differences in mouse strain, age, and stimulus conditions. Notably, the lack of sociability observed in adult BALB/c females in our study aligns with previous findings showing sex‐ and age‐dependent differences in this strain (Fairless et al. 2012). Second, while we observed significant microbial shifts alongside behavioral effects, we did not assess gut microbiota‐derived metabolites, which may influence brain development and function. Third, the study was not powered to detect correlations between individual behavioral outcomes and molecular or microbial markers. Fourth, although tissue dissections were performed consistently in the morning to minimize potential circadian variability, we did not include proteomic analyses, which could offer complementary insights into barrier function and cytokine regulation. Fifth, although this study was conducted in BALB/c mice—a strain known for low baseline sociability—our unpublished pilot data using C57BL/6 mice indicate that perinatal *L. reuteri* also enhances sociability in this strain. This suggests that the observed effects are not limited to a specific genetic background, which is consistent with previous animal studies using *L. reuteri* . Future studies addressing these limitations will be critical for elucidating the mechanistic pathways linking maternal probiotic supplementation to offspring brain and behavior.

In summary, our findings highlight that *L. reuteri* supplementation during early life exerts long‐lasting, sex‐specific effects on social behavior. Male and female offspring appear to engage distinct neuroimmune, neurodevelopmental, and potentially hormonal pathways in response to perinatal exposure. These differences may also involve epigenetic mechanisms, as the gut microbiota is known to influence DNA methylation, histone modification, and microRNA expression during early brain development, often in a sex‐dependent manner (Stilling et al. 2014). Elucidating these molecular pathways will be essential for designing effective, sex‐tailored microbial interventions. Ultimately, such insights could guide the development of precision microbiota‐based strategies to promote healthy brain development and reduce the risk of neurodevelopmental disorders such as ASD.

## Author Contributions

**Tatiana Siegler Lathrop:** formal analysis, investigation, methodology, project administration, visualization, writing – original draft, writing – review and editing. **Inés Martínez Sanchez:** formal analysis, investigation, project administration, visualization, writing – review and editing. **Ioannis S. Chronakis:** resources, supervision, writing – review and editing. **Rochellys Diaz Heijtz:** conceptualization, formal analysis, funding acquisition, investigation, project administration, resources, supervision, visualization, writing – original draft, writing – review and editing.

## Conflicts of Interest

The authors declare no conflicts of interest.

## Peer Review

The peer review history for this article is available at https://www.webofscience.com/api/gateway/wos/peer‐review/10.1111/jnc.70199.

## Supporting information


**Data S1:** jnc70199‐sup‐0001‐supinfo.docx.

## Data Availability

The data that support the findings of this study are available from the corresponding author upon reasonable request.
