# Supplementary material for: Sex‐Specific Long‐Term Effects of Perinatal Limosilactobacillus reuteri on Social Cognition, Gene Expression, and Gut Microbiota
Source: J Neurochem. 2025 Aug 19;169(8):e70199. doi: 10.1111/jnc.70199 (PMC12362329; doi:10.1111/jnc.70199)
Supplement: Supplementary file 1 — Data S1: jnc70199‐sup‐0001‐supinfo.docx. [file JNC-169-0-s001.docx]

**Supplementary Data**

**Sex-Specific Long-Term Effects of Perinatal *Limosilactobacillus reuteri* on Social Cognition, Gene Expression, and Gut Microbiota**

Tatiana Siegler Lathrop^1,^ Inés Martínez Sanchez^2^, Ioannis S. Chronakis^1^, Rochellys Diaz Heijtz^2^

^1^ Technical University of Denmark, DTU-Food, Research Group for Food Production Engineering, Laboratory of Nano-BioScience, Denmark

^2^ Department of Neuroscience, Karolinska Institutet, Stockholm Sweden

Tatiana Siegler Lathrop and Inés Martínez Sanchez contributed equally.

**Figure S1.** Experimental design. Pregnant dams received *L. reuteri* or control supplementation via drinking water from gestational day (GD) 6 to postnatal day (P) 7. Offspring were weaned at P21 and tested for adult behavior between postnatal days 70 and 84. Female offspring (10 weeks old) were tested first, followed by males (11 weeks old) the following week. Behavioral testing included the light–dark box (LDB; #1), three-chamber social approach task (3-CST; #2), and elevated plus maze (EPM; #3), with one rest day between each test. All animals were naïve to the procedures and were euthanized three days after the final behavioral test for tissue collection (prefrontal cortex, striatum, colon, and cecal content). Each experimental group included offspring from 4–6 litters, with no more than two pups of the same sex per dam.


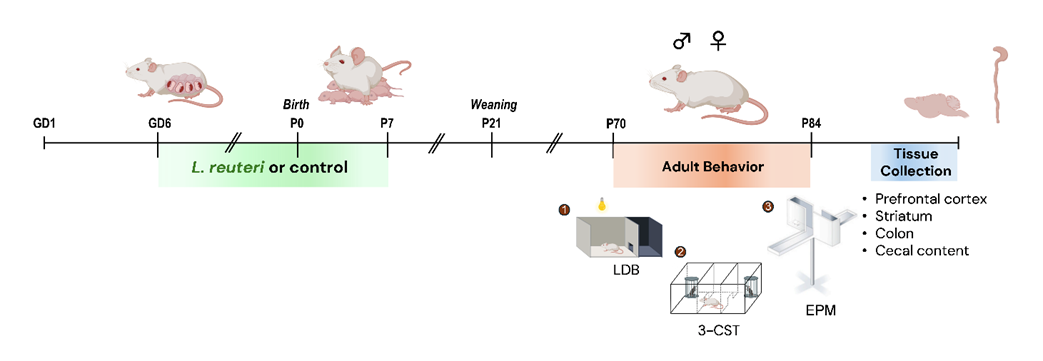


**Figure S2.** Effects of perinatal exposure to *L. reuteri* on offspring body weight at 3 and 10 weeks of age. (a) Body weights of male and female offspring at 3 weeks of age. (b) Body weights at 10 weeks of age. Bars represent mean ± SEM (in grams), n = 8 per group. As body weight data were not normally distributed in some groups (based on the Shapiro–Wilk test), comparisons between control and probiotic groups were made using the Mann–Whitney U test. At postnatal day 21 (3 weeks of age), probiotic-treated males showed significantly higher body weight compared to controls (U = 10, *P* = 0.0079), while no differences were observed in females (U = 25.5, *P* = 0.6737). At 10 weeks of age, male mice in the probiotic group also had higher body weight (U = 13, *P* = 0.0493), whereas female body weight did not differ significantly (U = 24.5, *P* = 0.4665). ** *P* < 0.01; * *P* < 0.05.


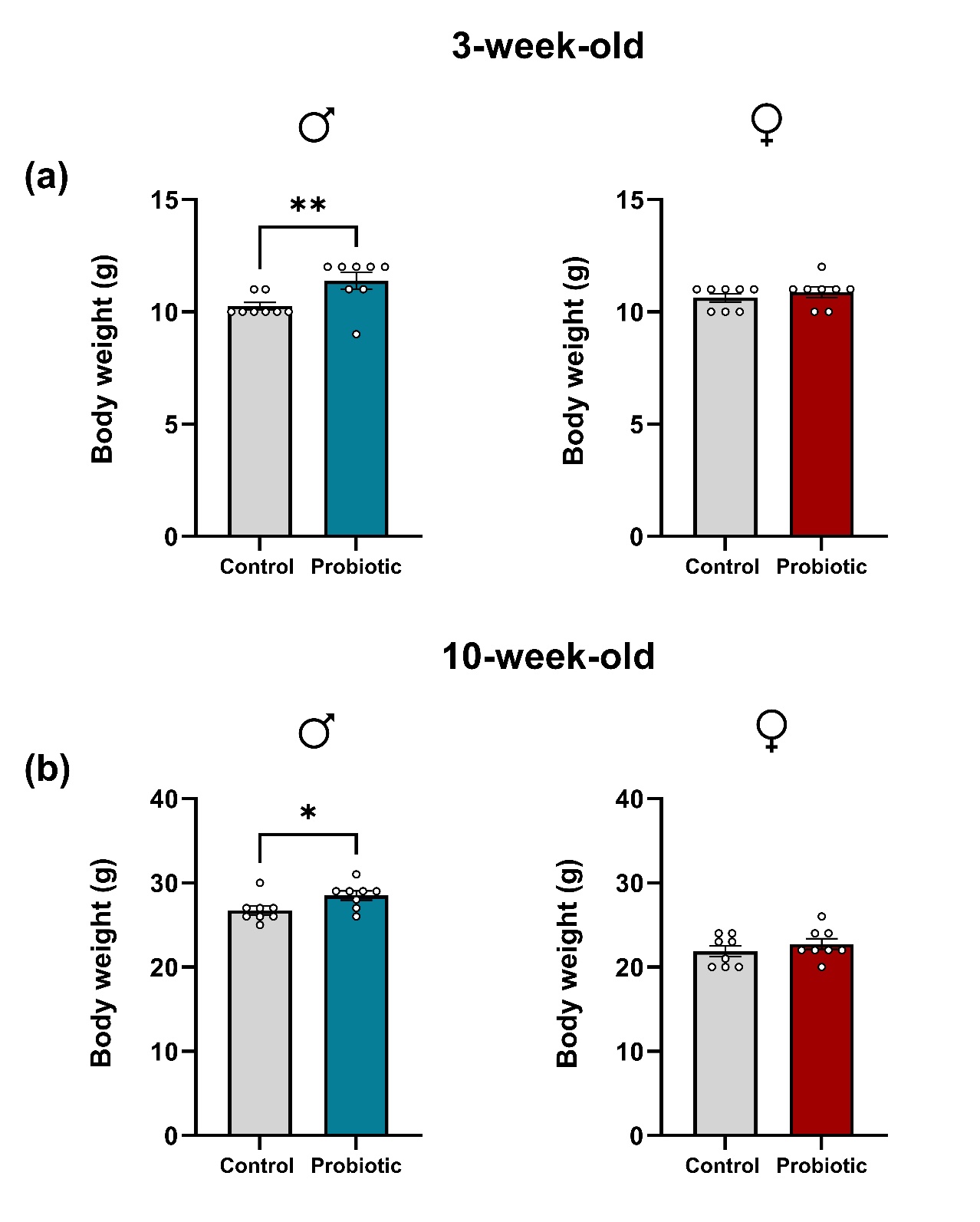


**Table S1.** Key resources table.

|  |  |  |  |  |
| --- | --- | --- | --- | --- |
|  |  |  |  |  |

| Resource Type | Name / Description | Supplier / Source | Catalog / Product No. | RRID |
| --- | --- | --- | --- | --- |
| Mouse strain | BALB/cJRj | Janvier Labs | — | SCR_026051 |
| Animal facility | Comparative Medicine Biomedicum | Karolinska Institutet | — | SCR_001548 |
| Mouse cage | Makrolon® Type III Polycarbonate Cage | Animalab | 031200256 | — |
| Software | GraphPad Prism v9 | GraphPad | — | SCR_002798 |
| Equipment | Acti-Mot detection system | TSE, Bad Homburg | STEMRALEPO0008YJL | — |
| Equipment | Elevated Plus Maze apparatus | Kinder Scientific | C080476212 | — |
| Software | EthoVision XT v11 | Noldus | NDS-NSE-EV-BASE | — |
| Extraction kit | QIAGEN RNeasy® Mini Kit | Qiagen | — | SCR_008539 |
| Equipment | TissueLyser II | Qiagen | — | SCR_018623 |
| Equipment | NanoDrop 2000C Spectrophotometer | Thermo Fisher | — | SCR_020309 |
| Extraction kit | iScript™ cDNA Synthesis Kit | Bio-Rad | — | SCR_008426 |
| Equipment | QuantStudio™ 7 Real-Time PCR System | Applied Biosystems, Life Technologies | — | SCR_020245 |

**Table S2.** Primer sequences used for quantitative real-time PCR (qRT-PCR), listed in the 5′ to 3′ direction.

| Name | Forward primer | Reverse primer | Ascension number |
| --- | --- | --- | --- |
| Ppia | agcatacaggtcctggcatc | ttcaccttcccaaagaccac | NM_008907.2 |
| Hprt | aagacttgctcgagatgtcatgaa | atccagcaggtcagcaaagaa | NM_013556.2 |
| Gapdh | agcttgtcatcaacgggaag | tttgatgttagtggggtctcg | NM_001289726.1 |
| Avpr1a | gtccatcagatttatccctgtctc | cacaagccccgtccaag | NM_016847.2 |
| Avpr1b | ctctccgtcttagccttaacctca | ctccatccacctgctccaaat | NM_011924.2 |
| Bdnf | gaagttggcttcctagcggt | taggccatgttgccttgtcc | NM_001048139.1 |
| Itgam (Cd11b) | aagcagctgaatgggaggac | tagatgcgatggtgtcgagc | NM_001082960.1 |
| Cldn3 | cctcatcgtggtgtccatcc | cgccaacaggaaaagcactc | NM_009902.4 |
| Cx3cr1 | gagcatcactgacatctacctcc | agaaggcagtcgtgagcttgca | NM_009987.4 |
| Ppp1r1b (DARPP-32) | agcacctgcagaccattagc | ttccatctctctggggctca | NM_144828.1 |
| Aif1 (Iba-1) | cctgaggagatttcaaaagctga | gtttggacggcagatcctca | NM_019467.2 |
| Il6 | gggactgatgctggtgacaa | tggtcttggtccttagccac | NM_031168.1 |
| Il10 | tgctgcctgctcttactgac | ttctgggccatgcttctctg | NM_010548.2 |
| F11r (JAM-A) | agctactctagagggggagc | cccagtggcacattttgtgg | NM_172647.2 |
| Mag | gatgccctcgaccatctcag | gtgggcttccaaggtgcata | NM_010758.2 |
| Mbp | tccatcgggcgcttctttag | gggatagcaggcctcgacag | NM_001025251.2 |
| Mog | agttggggatgaagcagagc | gcaccgaagtcttccctctc | NM_010814.2 |
| Muc3 | ccgatgtcaccacttctgct | gctctccaccagttcctcac | AF027131.1 |
| Ocln | ttccggccgccaaggt | tagcctcctggggatcaacc | NM_008756.2 |
| Oxtr | ctccacctacctgctgttgg | ttgacactactgacccgtgc | NM_001081147.1 |
| Slc15a1 (PepT1) | ggctcgattctacacctacatc | tgcccttcacatatttgtctgt | NM_053079.2 |
| Slc15a2 (PepT2) | ctagtcccccttttcactgtag | gcgtttcctttggactcattttt | NM_021301.4 |
| Slc46a2 | tcgctggtggagtatcagga | gagtaagagtccgatgcggg | NM_021053.4 |
| Slc46a3 | ctaacagggctcgagtgtcc | tgaacttcctcccggaatgc | NM_027872.4 |
| Trem2 | atgacacccttgctggaacc | gctagaggtgacccacagga | NM_001272078.2 |
| Syp | tcacaggcactaccaacgtc | atctacaggtctgtggggct | NM_009305.2 |
| Tjp1 (ZO-1) | gccttggcctagcatacaca | ggtaaggcattcctgctggt | NM_001252473.1 |
